# Association of COVID-19 coinfection with increased mortality among patients with *Pseudomonas aeruginosa* bloodstream infection in the Veterans Health Administration system

**DOI:** 10.1017/ash.2023.455

**Published:** 2023-12-15

**Authors:** Leila S. Hojat, Brigid M. Wilson, Federico Perez, Maria F. Mojica, Mendel E. Singer, Robert A. Bonomo, Lauren H. Epstein

**Affiliations:** 1 Department of Medicine, Division of Infectious Diseases, Case Western Reserve University, Cleveland, OH, USA; 2 Division of Infectious Diseases and HIV Medicine, University Hospitals Cleveland Medical Center, Cleveland, OH, USA; 3 Geriatric Research Education and Clinical Center (GRECC), the VA Northeast Ohio Healthcare System, Cleveland, OH, USA; 4 Case Western Reserve University, Cleveland VAMC Center for Antimicrobial Resistance and Epidemiology (Case VA CARES), Cleveland, OH, USA; 5 Research Service, VA Northeast Ohio Healthcare System, Cleveland, OH, USA; 6 Grupo de Resistencia Antimicrobiana y Epidemiología Hospitalaria, Universidad El Bosque, Bogotá, Colombia; 7 Departments of Pathology, Pharmacology, Molecular Biology and Microbiology, Biochemistry, and Proteomics and Bioinformatics, Case Western Reserve University School of Medicine, Cleveland, OH, USA; 8 Department of Population and Quantitative Health Sciences, Case Western Reserve University, Cleveland, OH, USA; 9 Infectious Diseases, US Department of Veterans Affairs Medical Center, Decatur, GA, USA

## Abstract

**Objective:**

*Pseudomonas aeruginosa* bloodstream infection (PA-BSI) and COVID-19 are independently associated with high mortality. We sought to demonstrate the impact of COVID-19 coinfection on patients with PA-BSI.

**Design:**

Retrospective cohort study.

**Setting:**

Veterans Health Administration.

**Patients:**

Hospitalized patients with PA-BSI in pre-COVID-19 (January 2009 to December 2019) and COVID-19 (January 2020 to June 2022) periods. Patients in the COVID-19 period were further stratified by the presence or absence of concomitant COVID-19 infection.

**Methods:**

We characterized trends in resistance, treatment, and mortality over the study period. Multivariable logistic regression and modified Poisson analyses were used to determine the association between COVID-19 and mortality among patients with PA-BSI. Additional predictors included demographics, comorbidities, disease severity, antimicrobial susceptibility, and treatment.

**Results:**

A total of 6,714 patients with PA-BSI were identified. Throughout the study period, PA resistance rates decreased. Mortality decreased during the pre-COVID-19 period and increased during the COVID-19 period. Mortality was not significantly different between pre-COVID-19 (24.5%, 95% confidence interval [CI] 23.3–28.6) and COVID-19 period/COVID-negative (26.0%, 95% CI 23.5–28.6) patients, but it was significantly higher in COVID-19 period/COVID-positive patients (47.2%, 35.3–59.3). In the modified Poisson analysis, COVID-19 coinfection was associated with higher mortality (relative risk 1.44, 95% CI 1.01–2.06). Higher Charlson Comorbidity Index, higher modified Acute Physiology and Chronic Health Evaluation score, and no targeted PA-BSI treatment within 48 h were also predictors of higher mortality.

**Conclusions:**

Higher mortality was observed in patients with COVID-19 coinfection among patients with PA-BSI. Future studies should explore this relationship in other settings and investigate potential SARS-CoV-2 and PA synergy.

## Introduction

Invasive infections caused by *Pseudomonas aeruginosa* (PA) are challenging to treat among hospitalized patients due to the organism’s high virulence and complex patterns of antimicrobial resistance, including decreased outer membrane permeability, efflux pump systems, and antibiotic-inactivating enzymes.^1–3^ The acquisition of resistance genes via horizontal transfer or spontaneous mutation can also induce additional mechanisms of resistance, either due to exposure to broad-spectrum antibiotics or nosocomial transmission.^4^ Mortality associated with PA is high, particularly in the bloodstream, with an estimated 30-d mortality rate for PA bloodstream infection (PA-BSI) between 24% and 32%.^5–7^

The Centers for Disease Control and Prevention (CDC) published encouraging data in 2019 indicating a decrease in multidrug-resistant (MDR) PA from 46,000 estimated cases and 3,900 deaths in 2012 to 32,600 cases and 2,700 deaths in 2017.^8^ However in 2022, CDC reported a 32% increase in rates of MDR PA from 2019 to 2020, largely attributed to antibiotic overuse in patients with COVID-19.^9^ In addition to observed higher rates of MDR PA, several studies have identified a recent rise in PA-BSI incidence. A retrospective analysis of English National Health Service acute trusts between August 2020 and February 2021 observed an increase both in incidence of hospital-onset PA-BSI (4.9–6.2 cases per 100,000 bed-days) and in percentage of patients with PA-BSI and concomitant COVID-19 infection (0.7–34.1% among PA-BSI cases).^10^ A study performed within the Ascension health system additionally noted a 28% increase in hospital-onset PA-BSI and approximately five times higher rate of PA-BSI among patients with COVID-19 compared to patients without COVID-19.^11^ Other published reports have demonstrated similar findings.^12,13^ While these studies collectively suggest that the rise in observed PA-BSI coincides with the onset of the COVID-19 pandemic, outcomes of patients with PA-BSI and COVID-19 coinfection have not been evaluated.

In this study, we aimed to use the Veterans Health Administration (VHA) population to study the impact of concomitant COVID-19 infection on all-cause mortality in patients with PA-BSI. We hypothesized that patients with PA-BSI and COVID-19 coinfection would have a higher risk of mortality compared with patients with PA-BSI only. Further, we hypothesized that patients with PA-BSI diagnosed during the COVID-19 pandemic period would have higher mortality than those with PA-BSI prior to this period, regardless of COVID-19 coinfection status.

## Materials and methods

### Data source and population

Clinical data were extracted from the VHA Corporate Data Warehouse (CDW), which serves as a repository for patient data recorded in the electronic health record for all patients throughout the VHA system. The VHA is the largest integrated healthcare system in the United States and provides care to a heterogeneous population of over 9 million Veterans distributed among both urban and rural settings throughout the country.^14,15^ Patients ≥18 yr with at least one hospital admission to a VHA facility as of January 1, 2006 were eligible to be included in this analysis. We defined a case of PA-BSI as a positive blood culture collected from a patient from January 1, 2009 through June 30, 2022. We excluded eligible cases 1) among patients with unknown mortality status 30 d from the index culture; 2) without hospitalization; and 3) in which the blood culture was collected greater than 7 d before or 30 d after the date of hospital admission. Multiple blood cultures with PA collected within 30 d from the same patient were included as one case. If a patient had multiple PA-BSIs that met our inclusion criteria collected more than 30 d apart, we only included the first episode of PA-BSI; thus, each case represented a unique patient. We defined the pre-COVID-19 period as January 1, 2009 through December 31, 2019 and the COVID-19 period as January 1, 2020 through June 30, 2022. Cases during the COVID-19 period were identified as having COVID-19 coinfection if a positive test result for COVID-19 was documented during the associated hospitalization.

We collected clinical information for each case-patient including age, sex, race, ethnicity, Charlson Comorbidity Index, and modified Acute Physiology and Chronic Health Evaluation (mAPACHE) score.^16^ mAPACHE score was based on clinical information collected within 48 h following the blood culture collection corresponding to the index PA-BSI occurrence for each case. Antimicrobial susceptibility testing results were obtained from laboratory reports within the CDW; individual laboratory susceptibility testing methods and standards were not available. We collected information regarding treatment against the identified PA isolate administered within 48 h of the index positive culture. Targeted treatment was defined as administration of an agent with in vitro activity against the identified PA isolate on at least one day between days 0 and 2 from the date of index positive blood culture collection.

We defined four resistance phenotype classifications: (1) difficult-to-treat resistant (DTR); (2) MDR; (3) other unclassified resistant (OUR); and (4) expected resistant (ER). DTR was defined as in vitro resistance or intermediate resistance to all tested agents within the beta-lactam and fluoroquinolone classes (and aztreonam if tested).^17^ DTR determination required in vitro testing results for ≥1 expanded-spectrum cephalosporin, ≥1 carbapenem, and ≥1 fluoroquinolone. MDR was assigned for isolates not meeting criteria for DTR and with in vitro resistance or intermediate resistance to ≥1 drug in at least three categories including piperacillins, expanded-spectrum cephalosporins, fluoroquinolones, carbapenems, and aminoglycosides.^18,19^ OUR was any other pattern of in vitro resistance not otherwise classified as DTR or MDR. We classified cases as ER if there was no resistance detected to any tested agent within the piperacillin, expanded-spectrum cephalosporin, fluoroquinolone, carbapenem, aminoglycoside, monobactam, or polymyxin classes.

### Analysis

We performed a descriptive analysis characterizing all PA-BSI cases by resistance phenotype, targeted antimicrobial treatment, and 30-d mortality rate by year. A multivariable logistic regression analysis was performed to characterize the independent association between the primary predictors of COVID-19 coinfection and COVID-19 period and the primary outcome of 30-d mortality, as defined by the odds ratio with 95% confidence interval (CI) estimates. Relative risk (RR) was calculated by modified Poisson regression using a sandwich estimator to model robust error variance.^20^ Models both with and without COVID-19 coinfection as a predictor were estimated. The pre-COVID-19 period was additionally divided into early (2009–2016) and late (2017–2019) periods in the model for better characterization of possible changing mortality trends over the study period. The late-pre-COVID-19 period (2017–2019) was limited to 3 yr to create a more comparable cohort to the 3-yr COVID-19 period (2020–2022). For categorical variables that were missing at least 15% or more of the observations, the missing values were recoded to a new category, “missing data.” Data for variables with a smaller proportion of missing observations were completed with multiple imputation using the mice package (version 3.15.0) with 10 imputations. Continuous predictor variables were rescaled (e.g., age in decades) or considered as categorical terms to allow for more interpretable assessment of effect size. Sensitivity analyses were performed using a complete case analysis and uncategorized continuous data. Due to concern that the estimated effect of targeted antimicrobial treatment administered within 48 h could be biased by death prior to this timepoint, analyses were also performed with and without targeted treatment within 48 h and mAPACHE score before and after excluding patients with death within 48 h. All analyses were performed in R version 4.1.2.

### Ethics

Data were obtained as part of a protocol approved by the Institutional Review Board of the Cleveland Veterans Affairs Medical Center to study MDR organisms in hospitalized patients.

## Results

### Characteristics of the study population

**Figure 1.**
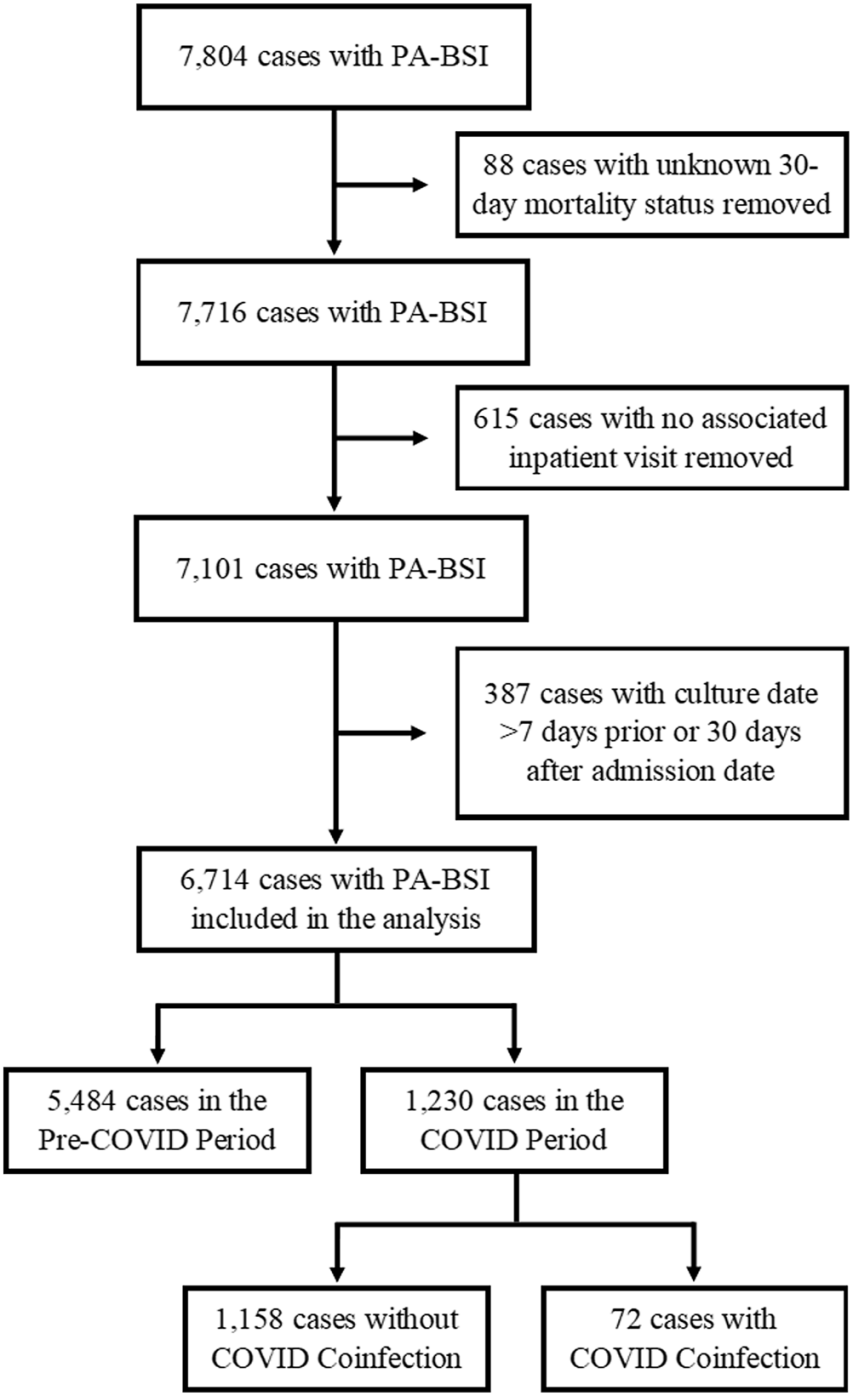
Flow diagram of inclusion and exclusion criteria resulting in 6,714 cases and further categorization into COVID-19 period and coinfection cohorts.

Among 7,804 PA-BSIs identified from January 1, 2009 to June 30, 2022, 6,714 PA-BSIs met our case definition (Figure 1). Among these, 5,484 (82%) were included from the pre-COVID-19 period, 1,158 (17%) were included from the COVID-19 period without COVID-19 coinfection, and 72 (1%) were included from the COVID-19 period with COVID-19 coinfection (Table 1). A majority of the case-patients were male, ≥70 yr old, white, and non-Hispanic, with multiple chronic comorbid medical conditions including uncomplicated diabetes mellitus (*n* = 2,892, 43.1%), cancer without metastases (*n* = 2,825, 42.1%), chronic pulmonary disease (*n* = 2,670, 39.8%), and chronic renal disease (*n* = 2,294, 34.2%).

**Table 1.** Demographic characteristics among patients with *Pseudomonas aeruginosa* bloodstream infection (*n* = 6,714) stratified by COVID-19 status and COVID-19 period

	Pre-COVID-19 period (*n* = 5484)	COVID-19 period		Total (*n* = 6714)
		COVID-19 negative (*n* = 1158)	COVID-19 positive (*n* = 72)	
Male sex, *n* (%)	5,346 (97.5)	1,125 (97.2)	71 (98.6)	6,542 (97.4)
Race, *n* (%)^a^				
White	3,885 (70.8)	808 (69.8)	47 (65.3)	4,740 (70.6)
Black	1,190 (21.7)	264 (22.8)	19 (26.4)	1,473 (21.9)
Other	64 (1.2)	16 (1.4)	0 (0)	80 (1.2)
Hispanic ethnicity, *n* (%)^b^	437 (8.0)	111 (9.6)	9 (12.5)	557 (8.3)
Age, median (IQR)	70 (63–79)	74 (68–79)	73 (66.8–83)	71 (64–79)
Charlson Comorbidity Index, median (IQR)	4 (2–6)	4 (2.3–7)	5 (3–7)	4 (2–6)
Modified APACHE score, median (IQR)	52 (41–65)	51 (40–63)	58 (44–71.5)	52 (41–65)
DTR phenotype, *n* (%)	188 (4.1)	18 (1.9)	0 (0)	206 (3.7)

Note. IQR, interquartile range; DTR, difficult-to-treat resistant.

a Excludes 421 patients with missing race data.

b Excludes 278 patients with missing ethnicity data.

### Antimicrobial resistance

**Figure 2.**
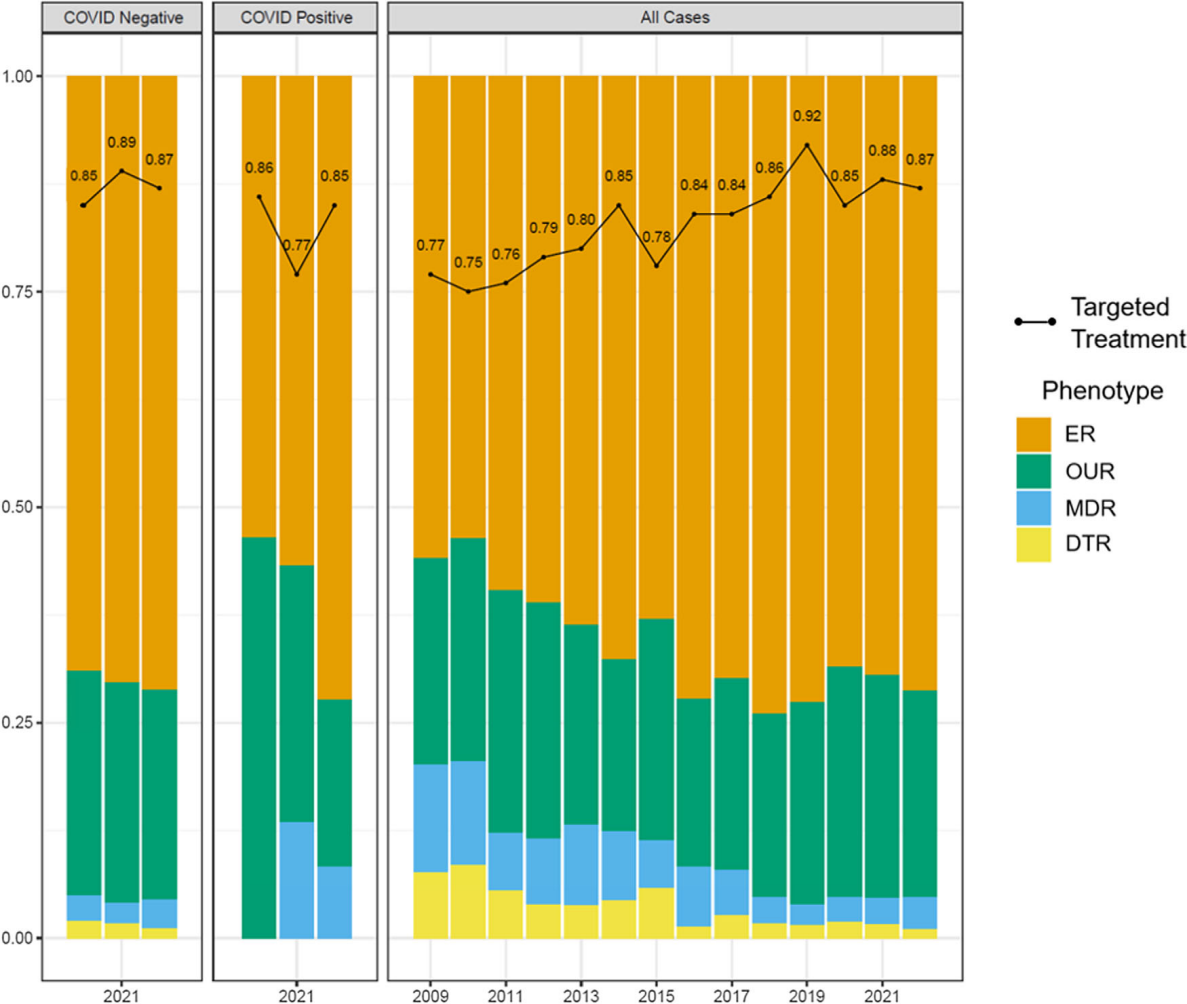
Figure demonstrates that the proportion of PA-BSI isolates within the VHA system susceptible to all tested agents increased over the study period, with a similar increase in proportion of all cases in which targeted treatment was administered within 48 h. A corresponding decrease in MDR and DTR resistance was also observed over the study period. Among patients with COVID-19 in addition to PA-BSI relative to patients with PA-BSI only, more isolates had OUR in 2020–2021 and more MDR was present in 2021–2022, but no DTR was present. Bar chart excludes 1,177 patients with missing resistance phenotype data. Line plot excludes 94 patients with missing data for targeted treatment within 48 h. 2022 data represent January 1 through June 30. PA-BSI, *Pseudomonas aeruginosa* bloodstream infection; VHA, Veterans Health Administration; ER, expected resistant; OUR, otherwise unclassified resistant; MDR, multidrug-resistant; DTR, difficult-to-treat resistant.

Antimicrobial susceptibility information sufficient for classification into the defined resistance phenotypes was available for 5,537 PA-BSI cases (82%). Throughout the study period, antimicrobial resistance decreased, with a downward trend observed for both MDR and DTR phenotypes and a steady increase in the proportion of PA isolates with ER; the proportion of PA isolates with OUR remained relatively unchanged (Figure 2). Among PA-BSI cases with COVID-19 coinfection, no DTR isolates were identified, though the proportion of MDR resistance in patients with COVID-19 was higher in 2021 and 2022 than in patients without COVID-19 coinfection during the same years.

Antimicrobial treatment data were available for 6,620 PA-BSI case-patients (99%). Throughout the study period, the proportion of case-patients who received targeted treatment for PA-BSI increased (Figure 2). The proportion of case-patients who received targeted treatment within 48 h was lowest among the DTR and MDR groups (20.6% and 53.4%, respectively) compared with the OUR and ER groups (78.4% and 89.8%, respectively).

### COVID-19 period and coinfection

**Figures 3.**
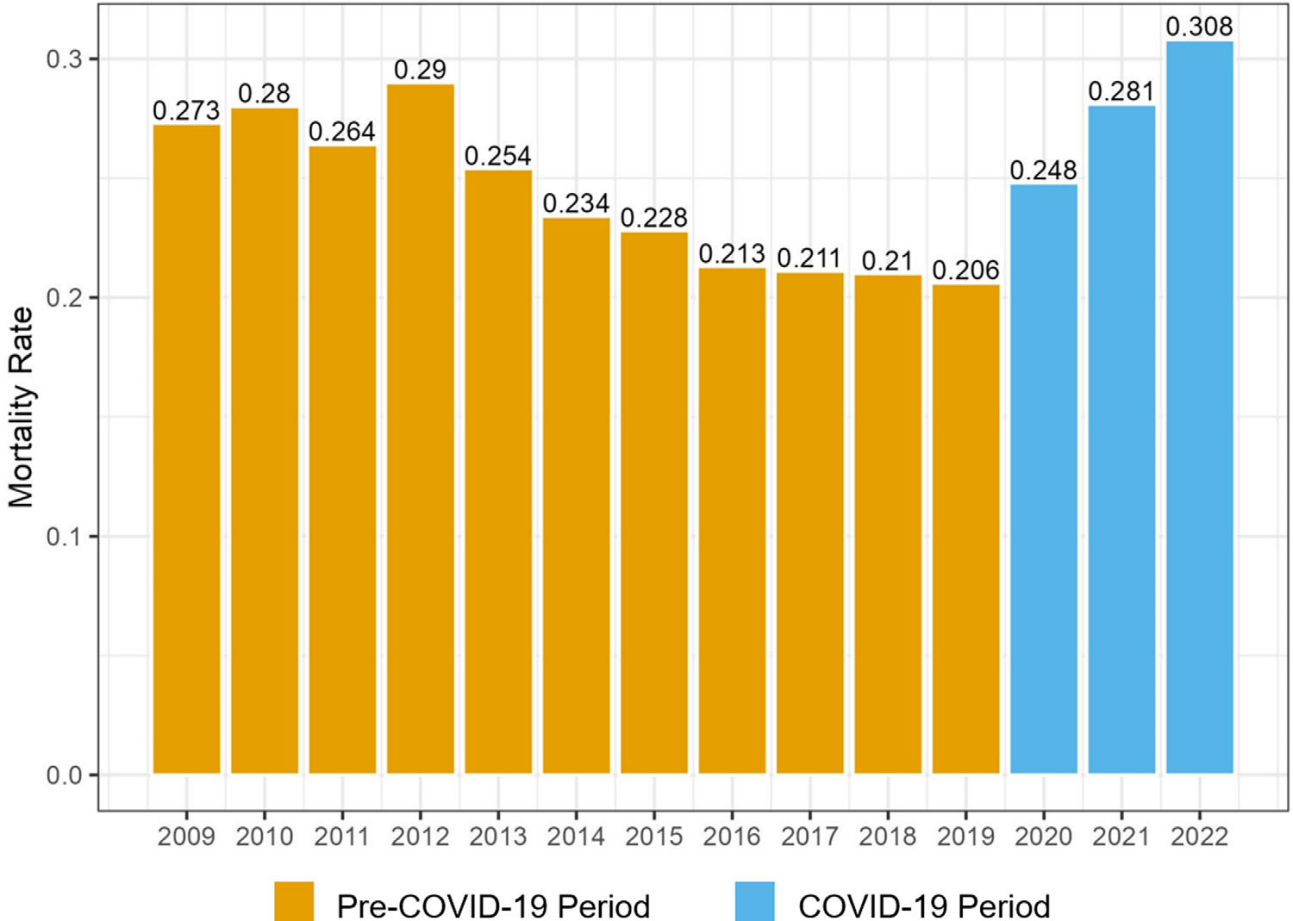
Figure indicates the proportion of PA-BSI cases in the VHA system meeting the primary end point of mortality at 30 d during each year of the study period. Mortality decreased across the pre-COVID-19 period to a low of 20.6% and increased during the COVID-19 period, peaking at 30.8% in 2022 through June 30. PA-BSI, *Pseudomonas aeruginosa* bloodstream infection; VHA, Veterans Health Administration.

**Figure 4.**
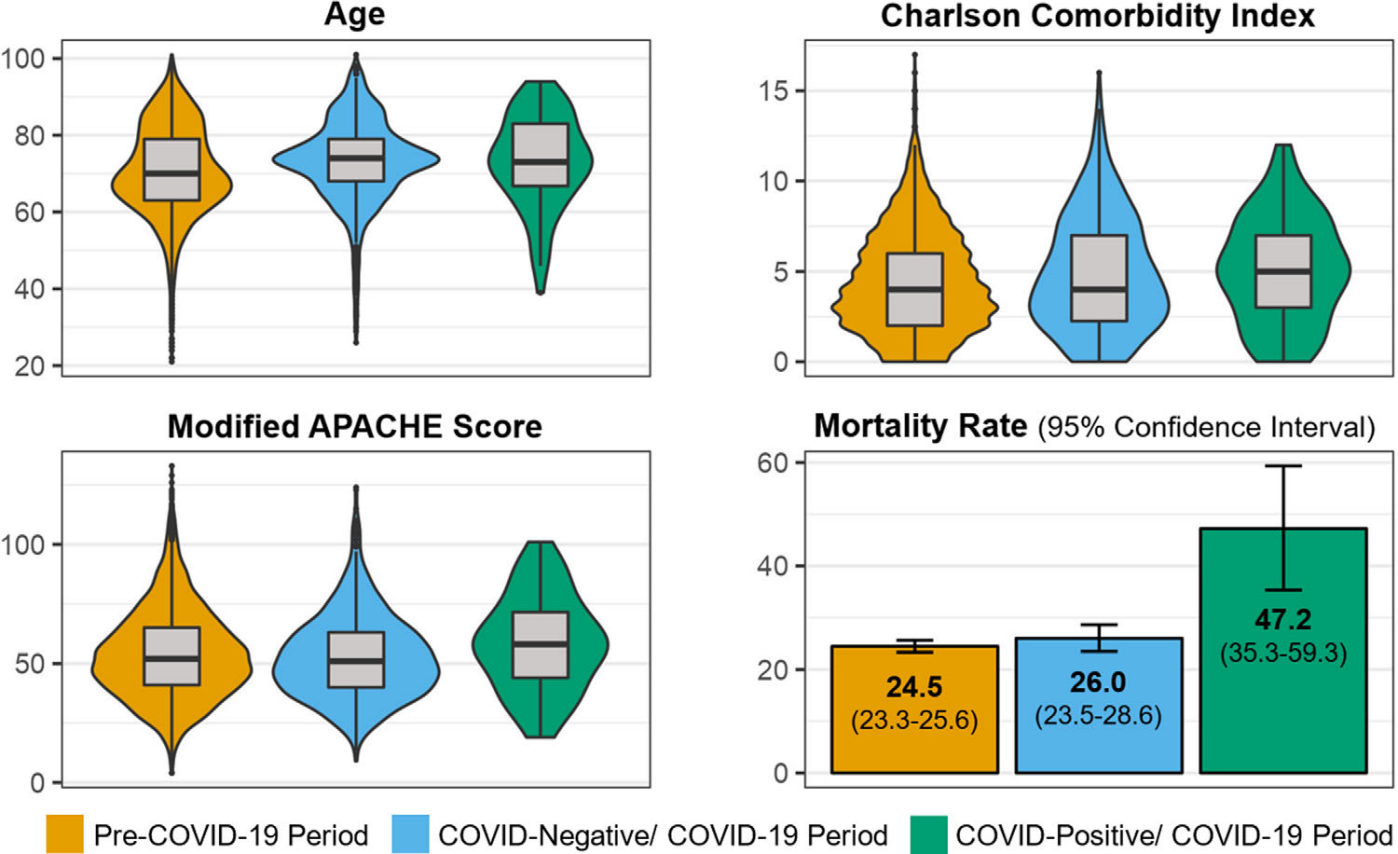
Distribution of clinical characteristics and mortality rate are shown stratified by COVID-19 period and COVID-19 coinfection status. Age **(A)**, Charlson Comorbidity Index **(B)**, and modified APACHE score **(C)** are noted to have a similar distribution across each cohort, despite that the mortality rate **(D)** is significantly higher in the COVID-19-positive/COVID-19 period patients.

Overall, 30-d all-cause mortality decreased among case-patients during the pre-COVID-19 period; conversely, we observed an increase in 30-d mortality among all case-patients during the COVID-19 period (not stratified by COVID-19 coinfection status) (Figure 3). Case-patients identified in either period were similar in terms of age, comorbidities, and severity of illness (characterized by the mAPACHE score) (Figure 4a–c). Case-patients with COVID-19 coinfection in the COVID-19 period had a 30-d mortality rate of 47.2%, whereas case-patients without COVID-19 during the COVID-19 period and case-patients in the pre-COVID-19 period had similar, lower mortality rates of approximately 25% (Figure 4d).

**Figure 5.**
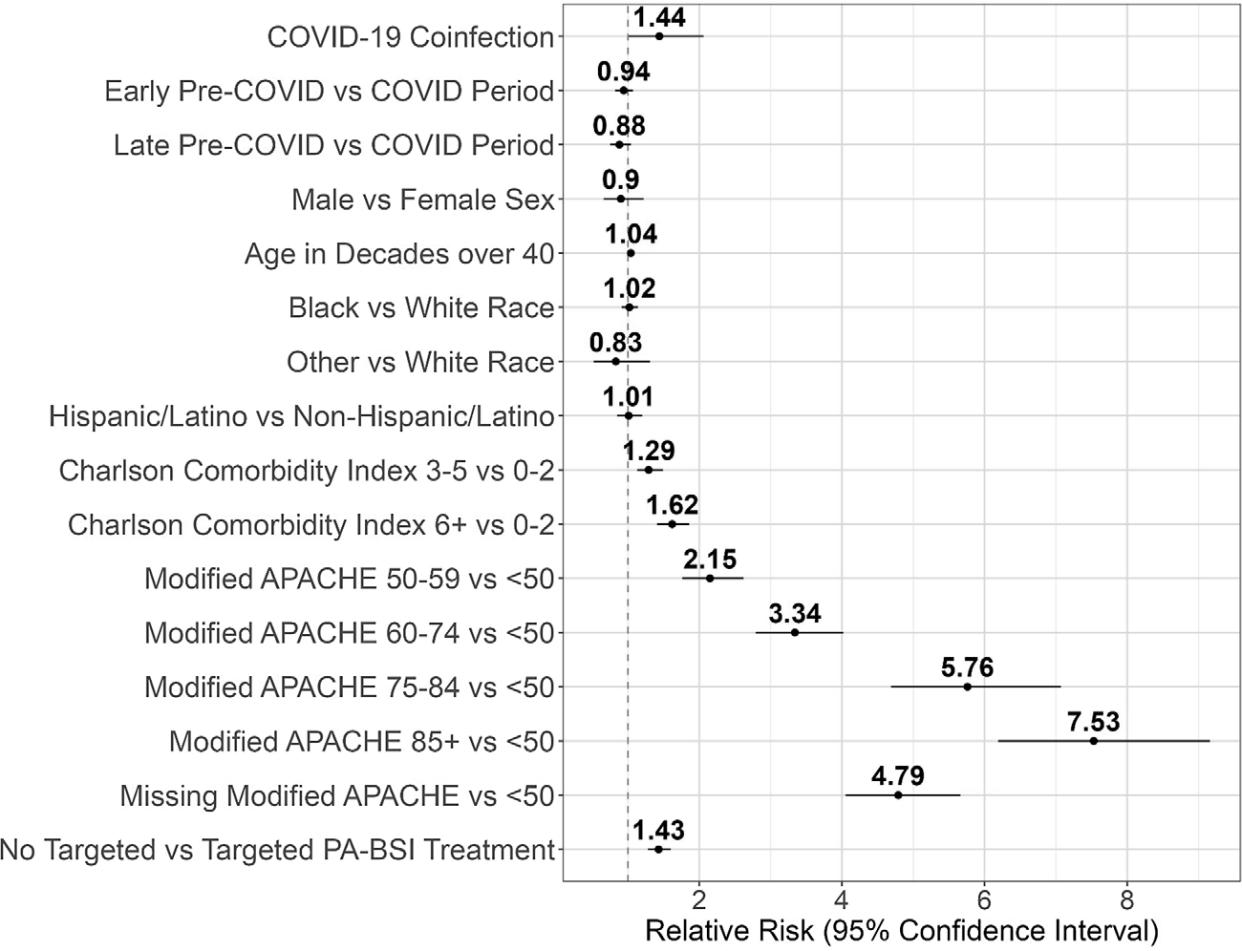
Modified Poisson analysis results illustrate the positive association between COVID-19 coinfection and increased all-cause 30-d mortality risk in patients with PA-BSI in the VHA system during the study period. Additional predictors associated with 30-d mortality include Charlson Comorbidity Index, administration of targeted treatment within 48 h, and modified APACHE score.

The pre-COVID-19 periods were associated with lower mortality among all patients with PA-BSI in the unadjusted analyses; however, this association was no longer significant after adjusting for the presence of COVID-19 coinfection (Table 2). After adjusting for potential confounders, patients with PA-BSI and COVID-19 coinfection were associated with higher mortality compared to patients with PA-BSI without COVID-19 in both the logistic regression (odds ratio 2.15, 95% CI 1.26–3.68) and modified Poisson (RR 1.44, 95% CI 1.01–2.06) models (Table 2). Higher Charlson Comorbidity Index, higher mAPACHE score, and lack of targeted treatment for PA-BSI within 48 h were also associated with higher risk of mortality in both analyses (Figure 5).

**Table 2.** Predictors of 30-d mortality among patients with *Pseudomonas aeruginosa* bloodstream infection (*n* = 6,714) with and without adjustment for COVID-19 coinfection

	Logistic regression				Modified Poisson			
	Unadjusted OR	95% CI	Adjusted OR	95% CI	Unadjusted RR	95% CI	Adjusted RR	95% CI
COVID-19 coinfection	NA	NA	2.15	1.26–3.68	NA	NA	1.44	1.01–2.06
Period								
COVID-19 period	Reference							
Early pre-COVID-19 period	0.85	0.72–0.99	0.89	0.76–1.05	0.91	0.80-1.03	0.94	0.82–1.07
Late pre-COVID-19 period	0.78	0.64–0.94	0.82	0.67–1.00	0.86	0.73–1.00	0.88	0.75–1.04
Sex								
Female	Reference							
Male	0.83	0.56–1.23	0.83	0.56–1.23	0.90	0.66–1.22	0.90	0.66–1.22
Age								
Age in decades over 40	1.07	1.01–1.13	1.07	1.02–1.13	1.04	1.00–1.09	1.04	1.00–1.09
Race								
White	Reference							
Black	1.03	0.88–1.20	1.03	0.88–1.20	1.02	0.91–1.15	1.02	0.91–1.14
Other	0.72	0.40–1.29	0.73	0.40–1.30	0.82	0.52–1.30	0.83	0.52–1.31
Ethnicity								
Non-Hispanic/Latino	Reference							
Hispanic/Latino	1.02	0.82–1.27	1.02	0.82–1.27	1.01	0.86–1.20	1.01	0.85–1.20
Charlson Comorbidity Index								
0–2	Reference							
3–5	1.45	1.23–1.72	1.45	1.23–1.72	1.29	1.13–1.49	1.29	1.13–1.49
6+	2.11	1.78–2.50	2.11	1.78–2.50	1.62	1.41–1.86	1.62	1.41–1.86
Modified APACHE score								
<50	Reference							
50–59	2.37	1.91–2.95	2.36	1.90–2.93	2.15	1.76–2.63	2.15	1.76–2.62
60–74	4.26	3.48–5.22	4.25	3.47–5.20	3.35	2.79–4.02	3.34	2.79–4.02
75–84	10.89	8.40–14.13	10.89	8.39–14.13	5.77	4.69–7.08	5.76	4.69–7.07
85+	22.13	16.78–29.19	21.95	16.63–28.96	7.59	6.24–9.23	7.53	6.19–9.16
Missing	7.52	6.22–9.08	7.48	6.19–9.04	4.80	4.06–5.68	4.79	4.05–5.66
Treatment administered within 48 h								
Targeted treatment	Reference							
No targeted treatment	1.84	1.59–2.13	1.84	1.59–2.13	1.43	1.29–1.60	1.43	1.28–1.60

Note. OR, odds ratio; CI, confidence interval; RR, relative risk.

In a sensitivity analysis, the DTR resistance phenotype was associated with higher mortality when the results were not adjusted for targeted antimicrobial treatment administered within 48 h (RR 1.32, 95% CI 1.10–1.58). Analyses including cases with no missing data, consideration of Charlson Comorbidity Index and mAPACHE as continuous variables, and exclusion of patients with death in less than 48 h did not substantially alter the relationship between the primary predictors and the primary outcome.

## Discussion

In this study of hospitalized adult patients with PA-BSI identified within the VHA system, we observed approximately 50% greater adjusted risk of 30-d all-cause mortality in patients with PA-BSI and COVID-19 coinfection compared with patients with PA-BSI only. Increased mortality was additionally observed in patients with higher Charlson Comorbidity Index, higher mAPACHE score, and no targeted antibiotic treatment administered within 48 h relative to patients without these features. The Charlson Comorbidity Index and mAPACHE score remained stable between the pre-COVID-19 and COVID-19 periods; however, the proportion of patients who received targeted treatment for PA-BSI increased. Decreasing rates of PA resistance were also observed throughout the full study period (2009–2022). While the mortality rate among case-patients increased during the COVID-19 period compared to the pre-COVID-19 period, the COVID-19 period itself did not appear to be associated with increased mortality independent of COVID-19 coinfection. This remained true even when isolating the late-pre-COVID-19 period corresponding to the 3 years immediately preceding the COVID-19 period with the lowest observed mortality rate of the entire study period. These results suggest that COVID-19 coinfection may account for the increased PA-BSI mortality observed during the COVID-19 period.

To our knowledge, our study is the first description of the direct relationship between COVID-19 and mortality in patients with PA-BSI. Our findings are consistent with previous reports of poor outcomes among hospitalized patients with any BSI type and COVID-19 coinfection. A multicenter study of adults with BSI and COVID-19 coinfection in 271 US facilities showed higher rates of intensive care unit admission (73% versus 55%), longer length of stay (35 d versus 26 d), and higher rates of ventilator use (53% versus 22%) compared with patients in the prepandemic period.^20^ Multiple studies have also identified higher mortality in patients with COVID-19 and BSI.^21–23^ Zhu et al. noted 27% higher mortality and 20 excess days of hospital stay in patients with hospital-acquired BSI during the COVID-19 pandemic period, regardless of COVID-19 coinfection status.^21^

Several factors could account for the increased mortality among patients coinfected with BSI and COVID-19. Excessive focus on the diagnosis of COVID-19 infection may delay recognition and workup of coexisting or secondary processes, thereby delaying initiation of appropriate treatment.^24^ Logistical barriers could affect management and subsequent outcomes in patients with COVID-19 related to isolation procedures and need for personal protective equipment.^25,26^ Additionally, circumstances at the institutional level related to the pandemic period, such as nursing- or provider-to-patient ratio, hospital volume, and availability of infectious diseases expertise, could impact outcomes on an individual level. Finally, the interaction of the two infectious processes could lead to a synergistic or additive effect on infectivity and the host response. Respiratory viruses have been found to alter pulmonary tissue innate immune function and promote bacterial growth through an increased burden on macrophages and decreased dendritic cell and macrophage antigen-presenting capabilities.^27,28^ The inflammation and fluids, which accumulate within the alveoli secondary to SARS-CoV-2 virus infection in particular, are thought to serve as good substrates for PA and other bacterial respiratory pathogens.^29^ Alteration of the respiratory tract microbiome may also play a role.^30^

Our study has several limitations. The retrospective study design generated potential biases, particularly for mAPACHE score and resistance phenotype. Missing data may have been secondary to factors undefined within our data set, such as intensive care unit admission and differences in antimicrobial susceptibility testing protocols between facilities. Other factors not included but potentially relevant to the primary outcome were source of PA-BSI, other concomitant infections, COVID-19 treatment, indication of recurrent versus initial COVID-19 infection, ventilatory support, facility complexity, and ratio of staff support to hospital volume. Focusing on PA-BSI may limit the generalizability of the results to other types of BSI, though this must be weighed against the potential risk of diluting the results with inclusion of other BSI types involving different patient populations, settings, sources of infection, and risk factors. The mAPACHE score may not be the best measure of disease severity in context of PA-BSI, but it is more readily available relative to other measures such as the Pitt bacteremia score, which rely on more resource-intensive data collection or natural language processing tools. The small number of COVID-19 positive cases also represented a potential limitation, and it is unknown whether patients in this study with detectable SARS-CoV-2 virus had a clinical syndrome consistent with COVID-19 infection, though asymptomatic COVID-19 infection could still pose immunologic effects. Finally, this study focused on the VHA population, which may not capture the full spectrum of patients at high risk for PA infection, such as individuals with cystic fibrosis.

There are also notable strengths. This is the first study to examine the impact of the COVID-19 pandemic on outcomes for patients with PA-BSI. The VHA CDW serves as a rich database for this study, capturing data from over 150 medical centers throughout the continental United States and Puerto Rico and spanning a period of almost 20 yr.^14^ Moreover, the VHA data regarding resistance trends generally align with CDC data in that rates of MDR PA infection were observed to decrease in the last decade until 2020 when an increase was observed; in our VHA cohort, the increase from 12 MDR/DTR cases in 2019 (2.6%) to 21 cases in 2020 (4.2%) represents approximately a 60% increase. Another strength is that while the subset of patients with concomitant COVID-19 infection was modest in size, this study included data for nearly 7,000 unique patients, thereby serving as one of the largest studied cohorts of patients with PA-BSI.

Our findings lay the groundwork for many potential future studies. The relationship between COVID-19 and PA should be explored in more detail to determine whether characteristics of either pathogen enhance the infectivity of the other or work synergistically against the host immune response, and inclusion of observations of patients with COVID-19 with and without PA-BSI could be helpful to this end. More features related to the COVID-19 period would also be worthwhile to investigate, such as impact of staffing, hospital volume, and resources on PA-BSI-associated outcomes. Utilizing alternative measures of disease severity and complexity such as Pitt bacteremia score, Sequential Organ Failure Assessment score, neutrophil-to-lymphocyte ratio, or the Veterans Health Administration COVID-19 Index (VACO) for COVID-19 mortality could enhance these types of analyses. Further, novel disease severity scores relevant to PA-BSI with inclusion of COVID-19 infection status should be validated for inclusion in future analyses. Finally, interventions that represent innovative measures to improve care and outcomes of patients with PA-BSI, including those with concomitant infections such as COVID-19, would be hugely impactful given the prevalence and poor outcomes associated with these conditions.

## Conclusions

Patients with PA-BSI had higher 30-d mortality during the COVID-19 period compared to the pre-COVID period. No targeted PA treatment within 48 h, higher mAPACHE score, and higher Charlson Comorbidity Index were also associated with higher mortality among all patients with PA-BSIs.
